# Comment on “Use of an elastic-scattering spectroscopy and artificial intelligence device in the assessment of lesions suggestive of skin cancer: A comparative effectiveness study”

**DOI:** 10.1016/j.jdin.2024.06.005

**Published:** 2024-08-16

**Authors:** Tai-Lin (Irene) Lee, Joe K. Tung

**Affiliations:** a Department of Radiology and Imaging Science, Emory University School of Medicine, Atlanta, Georgia; b Department of Dermatology, University of Pittsburgh School of Medicine, Pittsburgh, Pennsylvania; c Department of Dermatology, University of Pittsburgh Medical Center, Pittsburgh, Pennsylvania

**Keywords:** artificial intelligence, class imbalance, class balance, elastic-scattering spectroscopy, machine learning

*To the Editor:*

We read with great interest the article by Manolakos et al,^1^ which assesses the performance of an elastic-scattering spectroscopy (ESS) device to evaluate lesions suggestive of skin cancer. The authors used ESS to categorize clinically suspicious skin lesions into “monitor” (negative result) or “investigate further” (positive result) and compared the results with gross and histopathologic diagnoses by dermatologists and dermatopathologists. The device achieved high sensitivity in predicting all lesion types; however, the specificity was low across all major outcomes. The article did not provide information about the training data set used by the ESS algorithm, but the discrepancy between sensitivity and specificity suggests there might be some class imbalance in the underlying data set, likely skewed toward benign lesions. Imbalance data sets are not uncommon in health care associated data,^2^ but it could limit the generalizability of a machine learning model if class balance is significantly different in the test data set. Because the study participants in this article have a higher pretest probability, the differences are not amenable to posterior probability adjustment. This is confirmed by the fact that the model specificity is higher in unbiopsied benign lesions when compared with that of biopsied lesions. Although high sensitivity, even at the cost of lower specificity, is generally acceptable for screening tools, it is instrumental to apply this device algorithm on a patient population similar to the one it was originally trained on to ensure the suggested goal of prioritizing referrals and avoiding unnecessary procedures. It is possible to leverage the “spectral similarity score” to fine-tune cut-offs for positive predictions based on patients’ pretest probabilities.

In addition, representation of different skin types also needs to be considered when applying the device algorithm in the primary care setting. Of the authors’ 383 patients across testing and cross-validation groups, only one participant was non-White. It is well-known that artificial intelligence (AI) can perpetuate and exacerbate racial and ethnic disparities.^3^ Health care professionals report lower clinical confidence in managing skin conditions in dark skin tones.^4^ Any device utilizing an AI algorithm should be developed and cross-validated with this in mind to avoid exacerbating known disparities. Therefore, it is important to include more skin of color patients in research studies given that ESS patterns may vary across different skin tones.

Altogether, we emphasize the importance of applying AI algorithms to populations similar to the training data set and class balance. We should also make a concerted effort to address the underrepresentation of darker skin types and non-White populations in AI research to avoid inadvertently propagating racial bias in health care.^5^

## Conflicts of interest

None disclosed.
